# Association between pulse pressure and progression of chronic kidney disease

**DOI:** 10.1038/s41598-021-02809-8

**Published:** 2021-12-02

**Authors:** Toshiki Maeda, Soichiro Yokota, Takumi Nishi, Shunsuke Funakoshi, Masayoshi Tsuji, Atsushi Satoh, Makiko Abe, Miki Kawazoe, Chikara Yoshimura, Kazuhiro Tada, Koji Takahashi, Kenji Ito, Tetsuhiko Yasuno, Toshitaka Yamanokuchi, Kazuyo Iwanaga, Akiko Morinaga, Kaori Maki, Tamami Ueno, Kousuke Masutani, Shigeaki Mukoubara, Hisatomi Arima

**Affiliations:** 1 Department of Preventive Medicine and Public Health, Faculty of Medicine, Fukuoka University, 7-45-1 Nanakuma, Jonan-ku, Fukuoka, 814-0180 Japan; 2 Department of Internal Medicine, Division of Nephrology and Rheumatology, Faculty of Medicine, Fukuoka University, Fukuoka, Japan; 3 Department of Research Planning and Information Management, Fukuoka Institute of Health and Environmental Sciences, Fukuoka, Japan; 4 Department of Lifestyle and Welfare Information, Kindai University Kyushu Junior College, Fukuoka, Japan; 5 Department of Physical Therapy, Faculty of Medical Science, Fukuoka International University of Health and Welfare, Fukuoka, Japan; 6 Department of Internal Medicine, Nagasaki Prefecture Iki Hospital, Nagasaki, Japan

**Keywords:** Cardiology, Medical research, Nephrology

## Abstract

The aim of this study was to investigate the association between pulse pressure (PP) and chronic kidney disease (CKD) progression among the general population in Japan. We conducted a population-based cohort study of the residents of Iki Island, Nagasaki, Japan, from 2008 to 2018. We identified 1042 participants who had CKD (estimated glomerular filtration rate(eGFR) < 60 mL/min/1.73 m^2^ or the presence of proteinuria) at baseline. Cox’s proportional hazard model was used to evaluate the association between PP and progression of CKD. During a 4.66-year mean follow-up, there were 241 cases of CKD progression (incident rate: 49.8 per 1000 person-years). A significant increase existed in CKD progression per 10 mmHg of PP elevation, even when adjusted for confounding factors [adjusted hazard ratio 1.17 (1.06–1.29) p < 0.001]. Similar results were obtained even after dividing PP into quartiles [Q2: 1.14 (0.74–1.76), Q3: 1.35 (0.88–2.06), Q4: 1.87 (1.23–2.83) p = 0.003 for trend]. This trend did not change significantly irrespective of baseline systolic or diastolic blood pressures. PP remained a potential predictive marker, especially for eGFR decline. In conclusion, we found a significant association between PP and CKD progression. PP might be a potential predictive marker for CKD progression.

## Introduction

Chronic kidney disease (CKD) is characterized by renal function decline and/or the presence of albuminuria^1^. CKD is an important and established risk factor for end-stage renal disease (ESRD) and renal replacement therapy^2,3^. CKD also leads to cardiovascular disease or mortality^4,5^. Patients with CKD incur tremendous health care costs^6,7^; thus, CKD is a highly prioritized public health issue.

Some conditions such as high blood pressure^8–10^, hyperglycemia^11–13^, and smoking^14,15^ are thought to be risk factors for CKD progression. Among them, high blood pressure is one of the most established prognostic factors for CKD progression^6–8^. Pulse pressure (PP), which is easily calculated by subtracting the diastolic blood pressure (DBP) from systolic blood pressure (SBP), is a potential marker for atherosclerotic diseases including cardiovascular disease^16–20^, heart failure^16,21^, and stroke^16^. It was suggested that PP might be a superior predictor for coronary heart disease compared with SBP and DBP^20^. Thus, PP might also detect CKD progression effectively. However, few studies have investigated PP as a risk factor for CKD progression, especially in a general population.

Thus, the aim of this study was to investigate the association between PP and CKD progression among the general population in Japan.

## Methods

### Study design and participants

The Iki epidemiological Study of atherosclerosis and chronic kidney disease (ISSA-CKD) is a population-based cohort study of the residents of Iki Island, Nagasaki Prefecture, Japan. The details of the project have been described elsewhere^22^. Briefly, this project was started in 2016 and designed to decrease ESRD in Iki Island, where the rate of those undergoing hemodialysis was previously the highest in Nagasaki Prefecture. We conducted an observational study using health check-up data to help prevent CKD and atherosclerosis disease in Iki Island. From 2008 to 2018, 8029 residents underwent annual health check-ups conducted in Iki City. After the exclusion of 1856 residents who attended only once, 253 who had missing serum creatinine values or urine findings, and 4878 who did not have CKD at baseline, 1042 participants who had CKD (estimated glomerular filtration rate (eGFR) < 60 mL/min/1.73 m^2^ or the presence of proteinuria) at baseline were included as study subjects in the analysis (Supplementary Fig. 1). This study was carried out in accordance with the Declaration of Helsinki. The data were anonymized and processed so that individuals could not be identified and individual privacy was maintained; thus, informed consent was waived according to the Next Generation Healthcare Infrastructure Law in Japan^23^. This study was approved by institutional review board [Fukuoka University Clinical Research & Ethics Centre; FU-CREC (2017M010)]. FU-CREC also confirmed waiver of informed consent.

### Definition of independent variables

At each health check, anthropometric measurements, questionnaires, and blood and urine samples were collected. We used these variables from the start of the follow-up. Blood pressure (BP) was measured in the right upper arm using mercury, automated, or aneroid sphygmomanometers with appropriately-sized cuffs after at least 5 min of rest in a sitting position, by trained staff. BP was measured twice and the mean of the two values was calculated. PP was calculated by subtracting the DBP from SBP. We also divided the PP into quartiles (first quartile of PP (Q1): ≤ 48, Q2: 49–56, Q3: 57–65, Q4: ≥ 66). The targeted BP was the recommended BP level (< 130/80 mmHg for those with diabetes or proteinuria and < 140/90 mmHg for others) according to the Japanese guidelines^24^. Plasma glucose levels were determined by an enzymatic method and the presence of diabetes was defined by a fasting glucose level ≥ 126 mg/dL, non-fasting glucose level ≥ 200 mg/dL, HbA1c (NGSP) ≥ 6.5%^25^, or use of glucose-lowering therapies. The Japanese Diabetes Society (JDS) used the JDS HbA1c instead of NGSP until the fiscal year 2012. We converted the JDS HbA1c into NGSP using the following formula: NGSP (%) = 1.02 × JDS (%) + 0.25%. Serum low-density lipoprotein (LDL) cholesterol, high-density lipoprotein (HDL) cholesterol, and triglyceride levels were determined enzymatically. Dyslipidemia was defined by LDL cholesterol ≥ 140 mg/dL, HDL cholesterol < 40 mg/dL, triglycerides ≥ 150 mg/dL^26^, or the use of lipid-lowering medications. Serum uric acid was determined using an enzymatic method and hyperuricemia was defined by uric acid ≥ 7 mg/dL^27^. Information regarding smoking habits was obtained using a standard questionnaire. Height and body mass were measured with the participant wearing light clothes without shoes, and the body mass index (BMI, kg/m^2^) was calculated. Obesity was defined as BMI ≥ 25 kg/m^2^^28^. Current smokers were defined as participants who had smoked 100 cigarettes or more or who had smoked continuously for more than 6 months at the baseline examination. We classified drinking as never/chance drinker and habitual drinker using a questionnaire. Serum creatinine levels were determined by an enzymatic method and eGFR was estimated using the formula of the Japanese Society of Nephrology, as follows: eGFR (mL/min/1.73 m^2^) = 194 × serum creatinine ^−1.094^ × age ^−0.287^ (× 0.739; if female)^29^. Urinary protein was examined using the dipstick method and a 1 + or higher score was regarded as indicating the presence of proteinuria^14^.

### Definition of outcome variables

**Figure 1:**
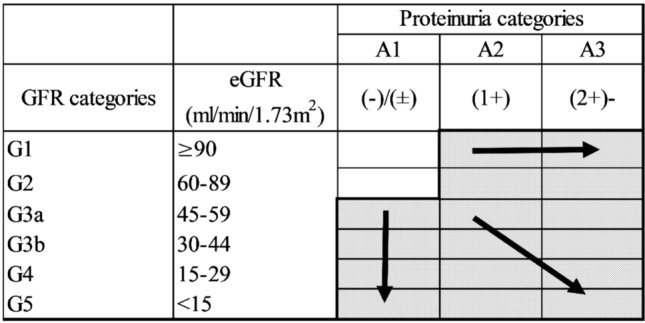
Definition of outcomes. The colored area was eligible for study (those with CKD at baseline). Progression of CKD was defined as exacerbation in the eGFR category or urinary protein category from baseline categories according to the Kidney Disease: Improving Global Outcome (KDIGO) CKD guidelines.

The primary outcome was progression of CKD among those with CKD at baseline. Progression of CKD was defined as exacerbation in the eGFR category or urinary protein category from baseline categories according to the Kidney Disease: Improving Global Outcome (KDIGO) CKD guidelines^1^, which was confirmed at the final follow-up examination, among participants who had CKD at baseline. eGFR categories were defined as G3a: eGFR 45–59, G3b: 30–44, G4: 15–29, and G5: < 15 mL/min/1.73 m^2^. Urinary protein was evaluated by urine dipstick and categorized as A1: ( −) or ( ±), A2: (1 +), and A3: (2 +) or more^5,30^. Progression of eGFR decline was exacerbation in the eGFR category from the baseline category (Fig. 1). Similarly, deterioration of urinary protein was defined as exacerbation in a protein category from the baseline categories (Fig. 1). We analyzed the progression of eGFR decline or proteinuria separately for further analyses.

### Statistical analysis

Baseline characteristics were compared across PP quartile categories using analysis of variance or the Kruskal–Wallis test for continuous variables and Pearson’s chi-square test for categorical variables. Follow-up was continued until the first episode of outcome, the end of the study period, or censoring. Incidence was calculated using the person-year approach. The influences of PP (per 10 mmHg) or PP quartile on the progression of CKD were estimated using Cox’s proportional hazards models. Covariates used in the analyses were sex, age, diabetes mellitus, dyslipidemia, hyperuricemia, obesity, smoking status (current or not current), drinking alcohol (habitual or never/chance), hemoglobin, and baseline eGFR and proteinuria (presence or absence). We then investigated the association between PP quartile and progression of CKD by stratifying SBP (SBP < 140 mmHg, SBP ≥ 140 mmHg) and DBP (DBP < 90 mmHg, SBP ≥ 90 mmHg). Next, we evaluated the association between PP and secondary outcomes including progression of eGFR decline and proteinuria separately. We compared the effect of PP quartile on the progression of CKD between subgroups (sex, age, diabetes mellitus, dyslipidemia, hyperuricemia, obesity, smoking status (current or not current), drinking alcohol (habitual or never/chance), hemoglobin, and baseline eGFR and proteinuria) by adding interaction terms to the models. We used the median age, hemoglobin, and baseline eGFR, and binary values of urinary protein (absent or present), for the subgroup analysis. Finally, we compared the discrimination of risk prediction between model 1 (< targeted BP or ≥ targeted BP), model 2 (PP ≥ 66 mmHg), and model 3 (combination of PP (< 66 or ≥ 66 mmHg and/or targeted BP) using Harrell’s C-index^31^. We used a cut-off point of PP 66 mmHg in model 3 because CKD progression was significantly increased at the point of Q4 (PP ≥ 66 mmHg), which was similar to the cut-off of a previous study (PP > 67 mmHg)^21^. Because there was high multicollinearity between continuous PP and continuous SBP (r = 0.801), we did not enter PP and SBP into one equation simultaneously. We found that 12.0% of the data on drinking, 0.10% on smoking status, 0.19% on BP-lowering medication, 0.38% on uric acid, and 0.76% on hemoglobin were missing. Therefore, we conducted a complete-case analysis in the primary analysis, followed by a sensitivity analysis using multiple imputation. Twenty datasets were created for multiple imputation. We regarded missing patterns as arbitrarily missing and used the Multiple Imputation by Chained Equation. STATA release 16 (STATA Corp, College Station, TX) was used for statistical analyses. All reported *P*-values were two-tailed, and the level of significance was set at *P* < 0.05.

### Ethics approval

This study was approved by the Fukuoka University Clinical Research & Ethics Centre (2017M010). The members of ethics committee that involved in the study were as follow; Fumihito Hirai, Shinichiro Yasunaga, Teruaki Izaki, Ryoko Sakuma, Satoshi Imaizumi, Kohichiro Kawashima, Toshiyasu Ikuta, Maho Oishi and Yuri Kusunose.

## Results

### Baseline characteristics

Baseline characteristics of the subjects are shown in Table 1. Of 1042 people who were eligible for this study, the number of people in Q1, Q2, Q3, and Q4 were 278 (26.7%), 260 (25.0%), 245 (23.5%), and 259 (24.9%), respectively. Mean age, SBP, FBS, the percentage of those taking BP-lowering medications, and those with diabetes and dyslipidemia were elevated in accordance with the elevation of PP quartile. In contrast, hemoglobin and eGFR tended to decrease with a larger PP. Regarding eGFR and proteinuria, those with a more advanced condition were more likely to be in the larger quartile of PP. There was no significant relationship between sex, DBP, TG, HDL-C, LDL-C, uric acid, hyperuricemia, BMI, obesity, smoking and drinking, and PP category.

**Table 1:** Baseline characteristics of study participants by pulse pressure quartile.

	Q1 (≤ 48)	Q2 (49–56)	Q3 (57–65)	Q4 (66 ≤)	Total	p-value
	N = 278	N = 260	N = 245	N = 259		
Age (years), mean ± SD	62.2 (9.0)	64.1 (6.5)	64.9 (5.8)	67.0 (5.4)	64.5 (7.1)	< 0.001
Sex, men (%)	160 (57.6)	122 (46.9)	123 (50.2)	133 (51.4)	538 (51.6)	0.093
Systolic BP (mmHg), mean ± SD	117.5 (12.2)	128.4 (10.4)	135.9 (11.7)	152.2 (15.8)	133.2 (18.0)	< 0.001
Diastolic BP (mmHg), mean ± SD	75.7 (11.0)	75.9 (10.1)	75.3 (11.4)	76.5 (11.8)	75.9 (11.1)	0.620
BP-lowering medication (%)	105 (37.8%)	113 (43.6%)	118 (48.4%)	147 (56.8%)	483 (46.4%)	< 0.001
Fast blood sugar (mg/dL), mean ± SD	96.5 (20.1)	99.2 (24.4)	103.1 (30.1)	104.4 (26.8)	100.6 (25.5)	0.010
HbA1c (%), mean ± SD	5.6 (0.9)	5.6 (0.8)	5.8 (1.0)	5.8 (1.0)	5.7 (0.9)	0.013
Diabetes (%)	30 (10.8%)	37 (14.2%)	53 (21.6%)	67 (25.9%)	187 (17.9%)	< 0.001
TG (mg/dL), median (Q25, Q75)	103.0 (74.0–147.0)	112.5 (79.0–154.0)	115.0 (79.0–161.0)	114.0 (83.0–163.0)	111.0 (78.0–155.0)	0.057
HDL-C (mg/dL), median (Q25, Q75)	59.0 (47.0–74.0)	57.0 (47.0–70.5)	57.0 (48.0–70.0)	56.0 (46.0–67.0)	57.0 (47.0–70.0)	0.180
LDL-C (mg/dL), median (Q25, Q75)	121.0 (103.0–141.0)	122.0 (99.5–143.0)	117.0 (100.0–146.0)	117.0 (98.0–141.0)	120.0 (100.0–142.0)	0.630
Dyslipidemia (%)	142 (51.1%)	161 (61.9%)	136 (55.5%)	156 (60.2%)	595 (57.1%)	0.049
Uric acid (mg/dL), median (Q25, Q75)	5.6 (4.6–6.8)	5.6 (4.8–6.5)	5.6 (4.7–6.7)	5.6 (4.8–6.7)	5.6 (4.7–6.7)	0.810
Hyperuricemia (%)	55 (19.9%)	43 (16.5%)	56 (23.1%)	54 (20.8%)	208 (20.0%)	0.310
BMI (kg/m^2^), mean ± SD	24.1 (3.8)	24.3 (3.4)	24.3 (3.5)	24.9 (3.8)	24.4 (3.6)	0.062
Obesity (%)	110 (39.6%)	105 (40.4%)	99 (40.4%)	121 (46.7%)	435 (41.7%)	0.310
**Smoking, n (%)**						
Current smoker	49 (17.6%)	34 (13.1%)	39 (16.0%)	34 (13.1%)	156 (15.0%)	0.370
**Alcohol, n (%)**						
Never/chance drinker (%)	198 (79.2%)	189 (79.7%)	162 (76.8%)	171 (78.1%)	720 (78.5%)	0.880
Habitual drinker (%)	52 (20.8%)	48 (20.3%)	49 (23.2%)	48 (21.9%)	197 (21.5%)	
Hemoglobin, mean ± SD	14.1 (1.6)	14.0 (1.4)	13.8 (1.4)	13.6 (1.6)	13.9 (1.5)	< 0.001
eGFR, mean ± SD	59.4 (15.8)	57.0 (12.4)	57.1 (13.3)	56.2 (16.2)	57.5 (14.6)	0.065
**KDIGO classification**						
eGFR categories						
G1 (eGFR ≥ 90)	67 (24.1%)	51 (19.6%)	46 (18.8%)	61 (23.6%)	225 (21.6%)	0.051
G2 (eGFR 60–89)	195 (70.1%)	187 (71.9%)	176 (71.8%)	164 (63.3%)	722 (69.3%)	
G3 (eGFR 30–59)	13 (4.7%)	20 (7.7%)	19 (7.8%)	22 (8.5%)	74 (7.1%)	
G4 (eGFR 15–29)	1 (0.4%)	1 (0.4%)	3 (1.2%)	6 (2.3%)	11 (1.1%)	
G5 (eGFR < 15)	2 (0.7%)	1 (0.4%)	1 (0.4%)	6 (2.3%)	10 (1.0%)	
Proteinuria categories						
A1 (proteinuria (−)/(±))	190 (68.3%)	191 (73.5%)	169 (69.0%)	157 (60.6%)	707 (67.9%)	0.011
A2 (proteinuria (+))	64 (23.0%)	49 (18.8%)	52 (21.2%)	59 (22.8%)	224 (21.5%)	
A3 (proteinuria (2+) ~)	24 (8.6%)	20 (7.7%)	24 (9.8%)	43 (16.6%)	111 (10.7%)	

*eGFR* estimated glomerular filtration rate, *KDIGO* Kidney Disease: Improving Global Outcome, *BMI* body mass index, *TG* triglyceride, *HDL-c* high-density lipoprotein cholesterol, *LDL-C* low-density lipoprotein cholesterol, *BP* blood pressure, *SD* standard deviation.

### Incidences and hazard ratios associated with pulse pressure and PP quartile in the progression of chronic kidney disease

**Figure 2:**
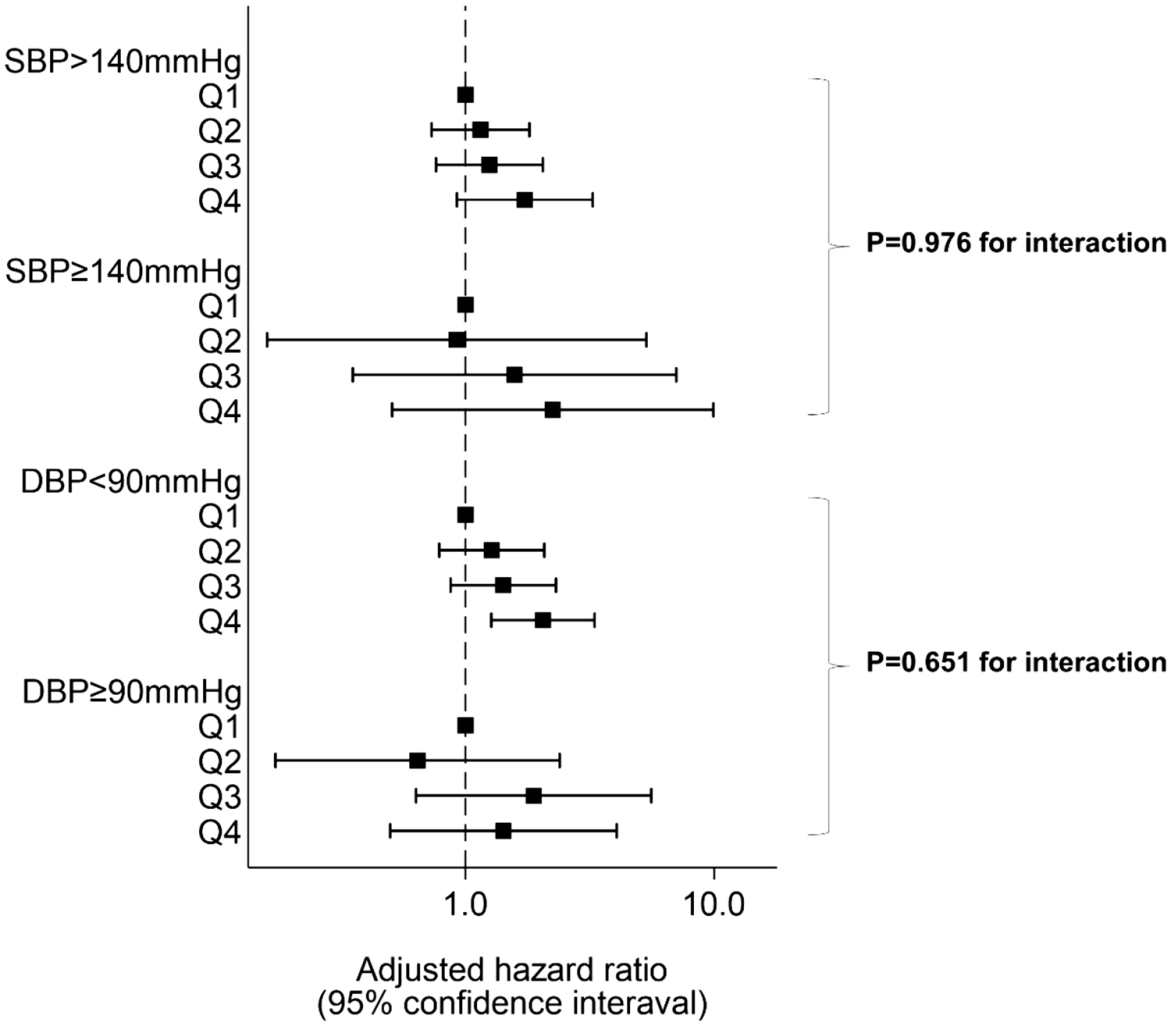
Stratified analyses of blood pressure: relationship between pulse pressure quartile and progression of CKD. Analyses were stratified by SBP (< 140, ≥ 140) and DBP (< 90, ≥ 90). Adjusted hazard ratios and 95% confidence intervals associated with pulse pressure quartiles for the progression of CKD were obtained, controlling for sex, age, diabetes mellitus, dyslipidemia, hyperuricemia, obesity, current smoking and drinking alcohol, and baseline eGFR and proteinuria. P for interaction was obtained by adding interaction terms to the models. Boxes and vertical lines represent hazard ratios and 95% confident intervals, respectively.

Incidences of the progression of chronic kidney disease are shown in Table 2. During a mean follow-up of 4.66 years (4855 person-years), there were 241 cases of CKD progression (incident rate: 49.8 per 1000 person-years). There was a significant increase in CKD progression per 10 mmHg of PP elevation, even when adjusted for confounding factors (adjusted HR 1.17 (1.06–1.29) p < 0.001). For each PP category, the incidence rates (per 1000 person-year) of Q1, Q2, Q3, and Q4 were 31.9, 40.5, 47.2, and 90.1, respectively. The crude HR of CKD progression increased linearly in accordance with larger PP [Q2: 1.27 (0.85–1.89), Q3: 1.47 (0.99–2.18), Q4: 2.72 (1.89–3.91) p < 0.001 for trend]. Similar linear results were obtained even after controlling for age, sex, BP-lowering medication, diabetes, dyslipidemia, hyperuricemia, obesity, smoking, drinking, hemoglobin, baseline eGFR, and proteinuria [Q2: 1.14 (0.74–1.76), Q3: 1.35 (0.88–2.06), Q4: 1.87 (1.23–2.83) p = 0.003 for trend]. There was no significant interaction between SBP, DBP, and PP for the progression of CKD (p = 0.976 for interaction between SBP (< 140 mmHg or ≥ 140 mmHg) and PP; p = 0.651 for interaction between DBP (< 90 mmHg or ≥ 90 mmHg) and PP) (Fig. 2).

**Table 2:** Incidences and hazard ratios associated with pulse pressure quartile for progression of chronic kidney disease.

	Incident rate (per 1000 person-years)	Crude HR (95% CI)	p-value	Adjusted HR	p-value
**Progression of CKD**					
PP (per 10 mmHg)	–	1.30 (1.20–1.42)	< 0.001	1.17 (1.06–1.29)	0.002
**PP quartile**					
Q1 (≤ 48)	31.9 (44/1379)	1.00 (reference)	< 0.001*	1.00 (reference)	0.003*
Q2 (49–56)	40.5 (54/1333)	1.14 (0.85–1.89)		1.14 (0.74–1.76)	
Q3 (57–65)	47.2 (55/1166)	1.47 (0.99–2.18)		1.35 (0.88–2.06)	
Q4 (≥ 66)	90.1 (88/977)	2.72 (1.89–3.91)		1.87 (1.23–2.83)	

*PP* Pulse Pressure, *CKD* Chronic kidney disease, *HR* Hazard ratio, *CI* confidence interval. Adjusted HRs were obtained, controlling for age, sex, BP-lowering medication, diabetes, dyslipidemia, hyperuricemia, obesity, smoking, drinking, and baseline eGFR and proteinuria.

*p for trend.

### Incidences and hazard ratios associated with pulse pressure quartile in the progression of GFR decline and proteinuria

**Figure 3:**
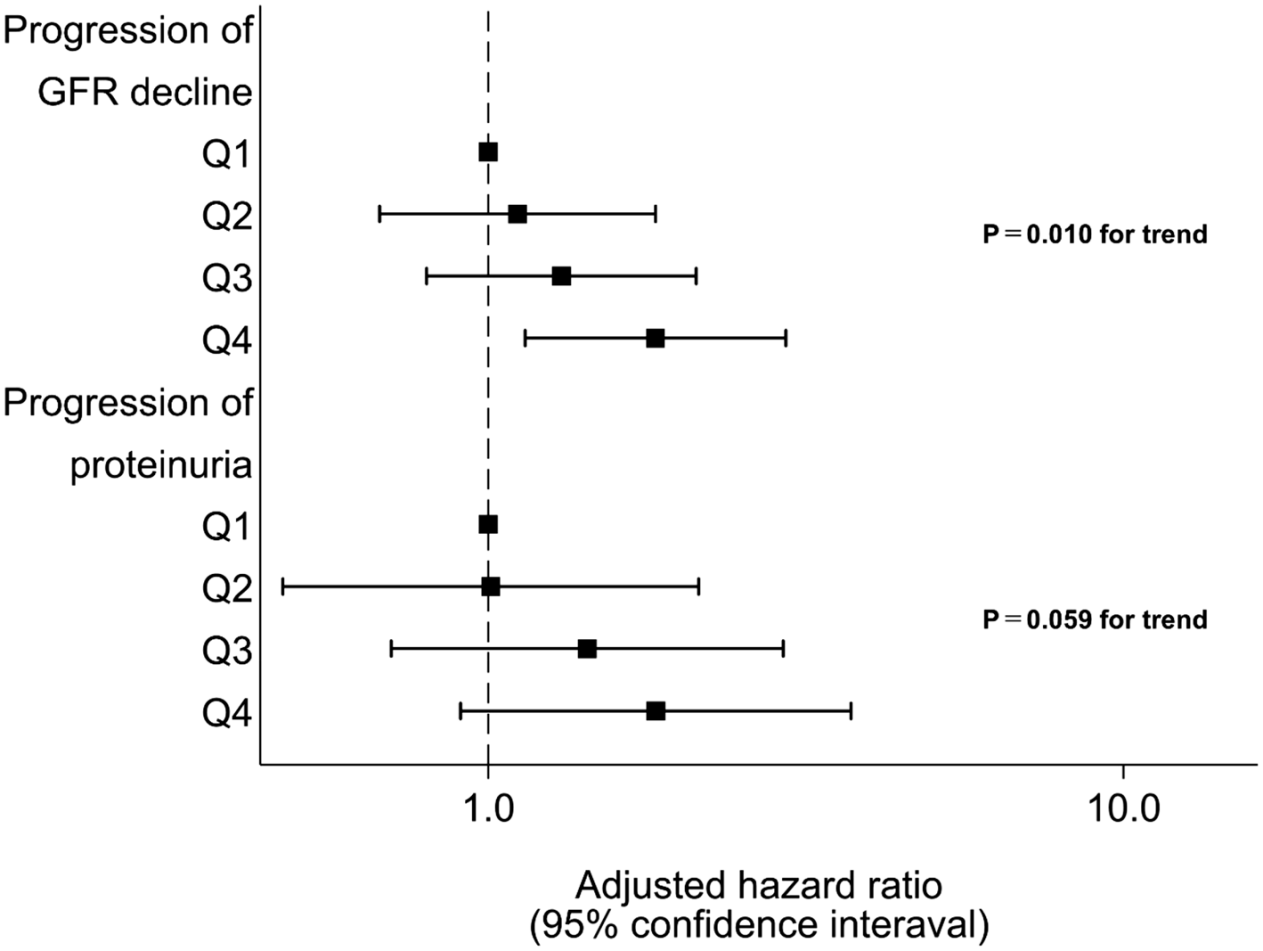
Effects of pulse pressure quartile on eGFR decline or progression of proteinuria. Adjusted hazard ratios and 95% confidence intervals associated with pulse pressure quartile for progression of eGFR decline or proteinuria were obtained, controlling for sex, age, diabetes mellitus, dyslipidemia, hyperuricemia, obesity, current smoking and drinking alcohol, and baseline eGFR and proteinuria. Boxes and vertical lines represent hazard ratios and 95% confident intervals, respectively.

We further separated the outcome of CKD into progression of GFR decline and proteinuria and analyzed their association with PP (Fig. 3). For the progression of GFR decline, there was a linear association with continuous PP and quartile of PP even after adjustment [continuous PP (per 10 mmHg); 1.17 (1.04–1.31) p = 0.007; quartile of PP: Q2: 1.11 (0.67–1.83), Q3: 1.30 (0.80–2.13), Q4: 1.83 (1.14–2.94) p = 0.010 for trend]. For proteinuria, there was a linear tendency between continuous PP and progression of proteinuria although statistical significance was not achieved [continuous PP (per 10 mmHg); 1.17 (0.98–1.40) p = 0.084], and similar results were obtained even after adjustment [quartile of PP; Q2: 1.01 (0.47–2.14), Q3: 1.43 (0.70–2.91), Q4: 1.84 (0.90–3.73) p = 0.059 for trend] (Fig. 3).

### Results of multiple imputation

Sensitivity analysis using the multiple imputation of missing values demonstrated comparable findings: Q2: 1.22 (0.82–1.84), Q3: 1.25 (0.83–1.88), Q4: 1.86 (1.27–2.74) for progression of CKD; Q2: 1.14 (0.72–1.82), Q3: 1.17 (0.74–1.85), Q4: 1.71 (1.10–2.63) for progression of GFR decline; Q2: 1.05 (0.52–2.12), Q3: 1.39 (0.71–2.72), Q4: 1.84 (0.95–3.57) for progression of proteinuria (Supplementary Table 1).

### Interaction between pulse pressure quartile and each subgroup in the progression of CKD

**Figure 4:**
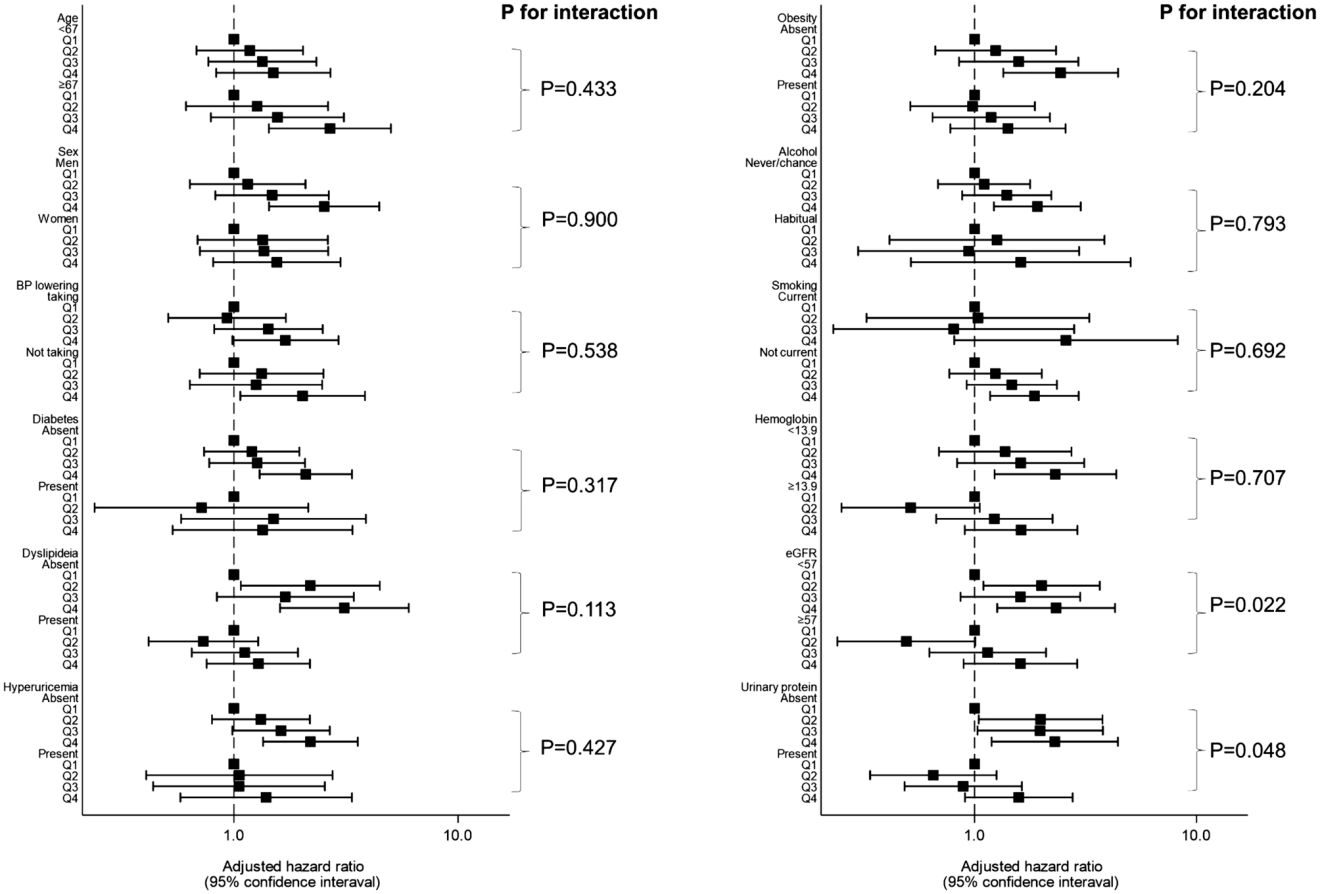
Subgroup analyses: interaction between pulse pressure and each covariate for the progression of CKD. Adjusted hazard ratios and 95% confidence intervals associated with pulse pressure quartile for the progression of CKD were obtained by adding interaction terms to Cox’s proportional hazards models, controlling for sex, age, diabetes mellitus, dyslipidemia, hyperuricemia, obesity, current smoking and drinking alcohol, hemoglobin, and baseline eGFR and proteinuria. P for interaction was obtained by adding interaction terms to the models. Boxes and vertical lines represent hazard ratios and 95% confident intervals, respectively.

Figure 4 shows the effect of PP quartile on the progression of CKD by subgroup. There were significant interactions between PP and baseline CKD (< 57, ≥ 57) (p = 0.022) or baseline urinary protein (absent or present) (p = 0.048). Statistically significant interactions were not detected for other subgroups (p > 0.1).

### Comparison of the discrimination of prediction between PP, targeted BP, and the combination of PP and targeted BP

Table 3 shows a comparison of the discrimination of prediction between model 1 (targeted BP), model 2 (PP ≥ 66 mmHg) and model 3 (combination of PP ≥ 66 mmHg and/or targeted BP). Harrel’s C-index for models 1, 2, and 3 was 0.7327 95% CI (0.6986–0.7668), 0.7356 95% CI (0.7010–0.7702), and 0.7330 95% CI (0.6988–0.7672), respectively. Dichotomous PP or adding dichotomous PP to targeted BP did not significantly improve the conventional targeted BP (differences between model 2 and model 1, and model 3 and model 1 were 0.0029 (− 0.0076–0.0135) p = 0.588 and 0.0003 (− 0.0043–0.0049) p = 0.854, respectively).

**Table 3:** Comparison of the discrimination of prediction for the progression of CKD between targeted BP, PP, and the combination of PP and targeted BP.

	Harrel’s C-index	Difference from model 1	p-value
Model 1	0.7327 (0.6986–0.7668)	–	–
Model 2	0.7356 (0.7010–0.7702)	0.0029 (− 0.0076–0.0135)	0.588
Model 3	0.7330 (0.6988–0.7672)	0.0003 (− 0.0043–0.0049)	0.854

Model 1: conventional targeted BP (< 130/80 mmHg for those with diabetes or proteinuria and < 140/90 mmHg); model 2: PP ≥ 66 mmHg; model 3: combination of PP (< 66 or ≥ 66) and/or targeted BP.

Other variables for adjustment were age, sex, BP-lowering medication, diabetes, dyslipidemia, hyperuricemia, obesity, smoking, drinking, and baseline eGFR and proteinuria.

## Discussion

In this study, we found a significant association between PP and progression of CKD. When PP was separated into quartiles, there was a significant increase in CKD progression at PP ≥ 66 mmHg, and CKD progression was also linearly increased in accordance with PP increase. This finding did not change significantly irrespective of baseline SBP or DBP. PP was still a potential predictive marker, especially for GFR decline, when we separated the outcome to progression of GFR decline and proteinuria. Although the discrimination of prediction for the progression of CKD was obtained with conventional targeted BP, there was no additional benefit of PP, or adding PP to the conventional targeted BP category.

PP is defined as the difference between the SBP and DBP, and is also determined by stroke volume and the compliance of large arteries^32^. A high PP has been well-established among elderly populations^33^ and those with diabetes^34^, CKD^35^, or ESRD^36^, and it also indicated large artery stiffness^20,37^ as a result of arteriosclerosis^38^. Previous studies reported PP was a potential marker for atherosclerotic diseases such as cardiovascular disease^16–20^, heart failure^16,21^, and stroke^16^. The present study revealed that PP is a potential risk marker for the progression of CKD. Individuals with high PP might already have stiff vessels, which could lead to microvascular kidney disease, or their BP might be uncontrolled because of low DBP. Furthermore, the kidney is a highly perfused organ with low resistance^39,40^. As aortic stiffness increases, indicated by increased PP, the kidney experiences greater pressure fluctuation and wave reflection, which leads to excessive pressure and flow pulsatility into the microvascular bed of the kidneys. Kidney arteries are subjected to high pulsate circumferential stress and high longitudinal shear stress^41^, which might cause microvascular ischemia and renal tissue damage^39^. Thus, increased PP, which is an index of arterial stiffness, might lead to glomerular hypertrophy, hyperfiltration, segmental glomerular sclerosis, and eventually, nephrosclerosis and fibrosis^39^.

The Framingham study reported PP was a stronger marker for coronary heart disease than BP^20,39^. However, a meta-analysis by the Japan Arteriosclerosis Longitudinal Study-Existing Cohort Combine Group reported that PP was a less important predictor for cardiovascular disease^42^. Our study revealed that although PP was still a potential predictive marker for CKD progression, there was no significant improvement in prediction when using PP or adding PP to conventional targeted BP. These differences in study findings might be related to the difference in outcomes, ethnicity, or study design. Another possibility might be differences in the statistical models used—our study did not enter PP and SBP simultaneously into one equation because of high multicollinearity. As an alternative, we used Harrel’s C-index to compare the discrimination of prediction for different models in contrast to previous studies that used the likelihood ratio test^20,39^.

The results of our subgroup analyses showed PP was affected by the extent of eGFR or proteinuria. Interestingly, PP seemed to be more related to CKD progression for those with lower eGFR or without proteinuria, suggesting proteinuria is a greater risk factor for CKD progression than PP. Another hypothesis is that two pathophysiologic mechanisms—ischemia and loss of autoregulation—are involved in hypertension-related renal disease^43,44^. Proteinuria is strongly related to hyper-infiltration caused by the loss of autoregulation^43^. However, increased PP might be closely related to parenchymal ischemia associated with the narrowed lumen of affected vessels rather than the loss of autoregulation^45^, which was closely related to GFR decline^33^. However, further studies to confirm this are needed.

Although this was a large-scale observational study of the general Japanese population, there were some limitations. First, because the study design was observational, we could not clarify a causal relationship. Second, the findings of the present analysis may be affected by selection bias because people with healthy lifestyles are more likely to attend health check-ups conducted by the local government than those with unhealthy lifestyles. Third, different sphygmomanometers were used, although BP was measured by trained staff according to standardized guidelines. Fourth, we did not determine competing risks, for example, death, because of data limitations. Finally, the accuracy of the dipstick method for the diagnosis of proteinuria is limited.

## Conclusion

We found a significant association between PP and progression of CKD. PP might be a potential predictive marker for the progression of CKD.

## Supplementary Information

Supplementary Information.

## Data Availability

The datasets used in this study are not publicly available due to the privacy policy of the data provider. However, the data are available from the corresponding author on reasonable request.
