# Supplementary material for: Association between pulse pressure and progression of chronic kidney disease
Source: Sci Rep. 2021 Dec 2;11:23275. doi: 10.1038/s41598-021-02809-8 (PMC8640028; doi:10.1038/s41598-021-02809-8)
Supplement: Supplementary file 1 — Supplementary Information. [file 41598_2021_2809_MOESM1_ESM.docx]

**Supplementary information**


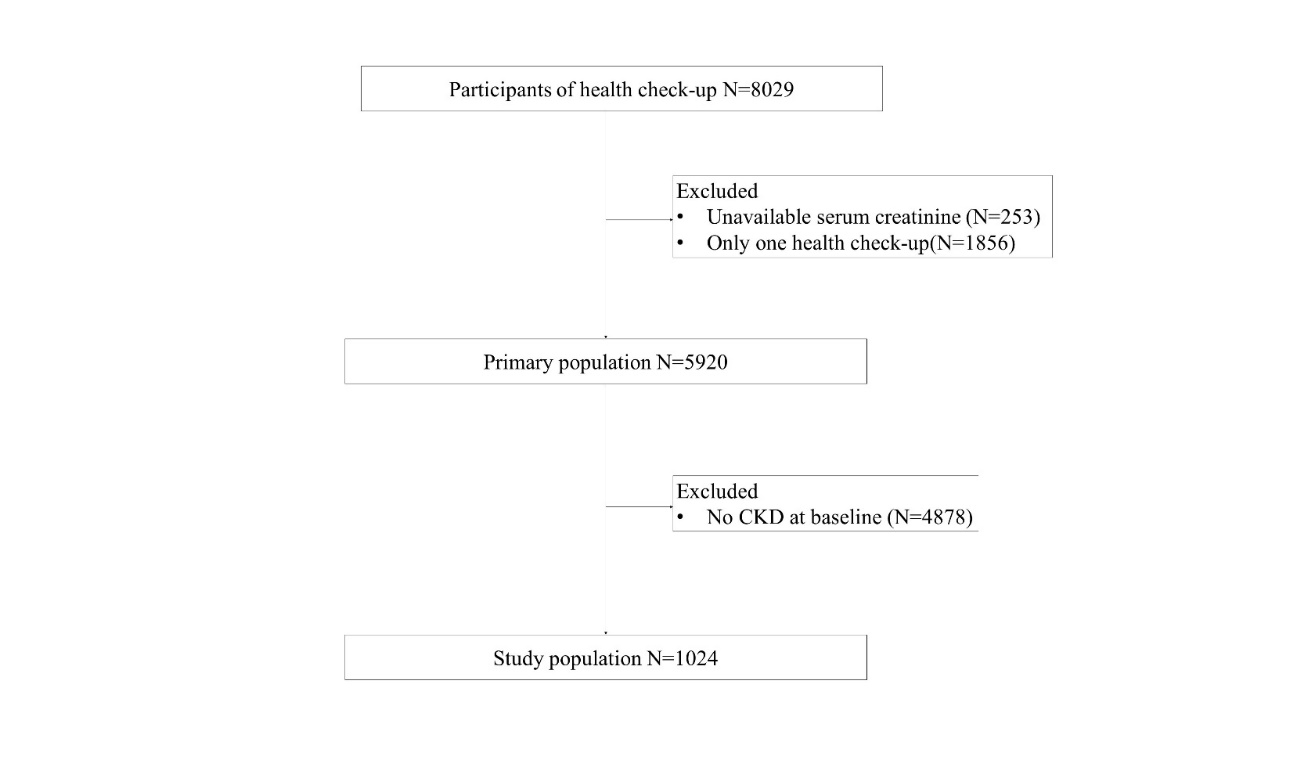


**Supplementary Figure1. Flow chart of participant selection**

**Supplementary table1. Result of multiple imputation**

|  | Adjusted HR (95% CI) | p-value |
| --- | --- | --- |
| Progression of CKD |  |  |
| Q1 | 1.00 (reference) | - |
| Q2 | 1.22 (0.82 to 1.84) | 0.327 |
| Q3 | 1.25 (0.83 to 1.88) | 0.279 |
| Q4 | 1.86 (1.27 to 2.74) | 0.002 |
| Progression of GFR decline |  |  |
| Q1 | 1.00 (reference) | - |
| Q2 | 1.14 (0.72 to 1.82) | 0.569 |
| Q3 | 1.17 (0.74 to 1.85) | 0.507 |
| Q4 | 1.71 (1.10 to 2.63) | 0.016 |
| Progression of albuminuria |  |  |
| Q1 | 1.00 (reference) | - |
| Q2 | 1.05 (0.52 to 2.12) | 0.900 |
| Q3 | 1.39 (0.71 to 2.72) | 0.331 |
| Q4 | 1.84 (0.95 to 3.57) | 0.071 |
